# Anesthetic management of a giant paraganglioma resection: a case report

**DOI:** 10.1186/s12871-022-01766-7

**Published:** 2022-07-11

**Authors:** WeiBing Wang, Hui Zhou, AiJiao Sun, JingBo Xiao, DongShu Wang, DaXiang Huang

**Affiliations:** 1 Department of Anesthesiology, The Affiliated AnQing Municipal Hospitals of Anhui Medical University, AnQing, China; 2 Department of Cardiovascularology, The Affiliated AnQing Municipal Hospital of Anhui Medical University, 352th, Renming Road, AnQing, 246003 China; 3 Department of General Surgery, The Affiliated AnQing Municipal Hospitals of Anhui Medical University, AnQing, China; 4 Department of Endocrinology, The Affiliated AnQing Municipal Hospitals of Anhui Medical University, AnQing, China

**Keywords:** Pheochromocytoma, Paraganglioma, Anesthesia, Giant, Catecholamine

## Abstract

**Background:**

Patients with pheochromocytomas are often diagnosed with acute myocardial infarction (AMI) due to initial symptoms of palpitations and chest tightness. We describe a case of AMI syndrome where a giant paraganglioma was unexpectedly identified. The anesthetic management of the paraganglioma resection was challenging and complex.

**Case presentation:**

A 66-year-old woman was admitted to the emergency department for complaints of palpitations, chest tightness and vomiting. A laboratory test revealed that troponin I and N-terminal pro-brain natriuretic peptide levels were dramatically increased. Emergency percutaneous coronary angiography (CAG) showed normal coronary arteries. In addition, the serum levels of free catecholamines were increased, and computed tomography and magnetic resonance imaging revealed a heterogenous mass lesion in the right retroperitoneal. All of this ultimately confirmed the diagnosis of pheochromocytoma. After three weeks of careful preoperative preparation by a multidisciplinary team, and an anesthesiologist team develops detailed perianesthesia management strategies to maintain hemodynamics and blood glucose stability and regulate acid–base balance, pheochromocytoma resection was performed successfully. About 2 weeks later, the patient was discharged healthy. A postoperative pathology test confirmed paraganglioma.

**Conclusions:**

To our knowledge, giant pheochromocytoma resection is a complex challenge for the anesthesiologists, this clinical case may supply a thoughtful experience for anesthetic management in the resection of giant pheochromocytomas. Adequate preoperative evaluation and prudent perianesthesia management by anesthesiologists are important guarantees for patients to obtain a good prognosis and discharge healthily.

## Background

Pheochromocytomas and paragangliomas (PPGLs) are rare tumors originating from the chromaffin tissue of the neuroectoderm. Most patients with these tumors are diagnosed with acute stress cardiomyopathy or acute coronary syndrome due to abnormally high catecholamine levels [1]. It is estimated that the incidence of PPGLs is approximately two to eight per million of the worldwide population [2]. Due to multiple patients’ diverse clinical symptoms and complex imaging manifestations, especially the potential metastasis of PPGLs, it is difficult to make a timely diagnosis of PPGLs [3]. The high levels of catecholamine is a serious threat to the lives of patients, and the size of PPGLs are positively correlated with the secretion levels of catecholamine. Meanwhile the levels of catecholamine are positively correlated with the maximum systolic blood pressure, which can reach the peak when the tumor is detected during the operation [4]. Surgical resection is the ultimate treatment for PPGLs, which is associated with a significant risk of mortality and morbidity. PPGLs require careful preoperative preparation and, perioperative management. The most important considerations are the regulation of hypertension, correction of vessel volume depletion, management of arrhythmia, improvement of cardiac function, correction of electrolyte disturbances and acid–base balance, and improvement of hyperglycemia [5]. All of these are critical strategies for reducing postoperative morbidity and mortality in patients with PPGLs.

## Case presentation

### Onset symptoms

A 66-year-old woman was admitted to the emergency department for complaints of palpitations, chest tightness, and vomiting. On admission, the patient was conscious and communicating well with doctors, and with a Glasgow Coma Scale 15, she complained of palpitations frequently occurring in the past decade, especially with right-sided holding objects or right-sided sleeping. The patient’s blood pressure was 168/105 mmHg, heart rate (HR)142 beats/minute, and with a peripheral capillary oxygen saturation (SpO_2_) of 96%. Emergency electrocardiography (ECG) showed an ST segment elevation of 0.8 mv in II, III, and aVF leads.

### Diagnostic process

**Fig. 1:**
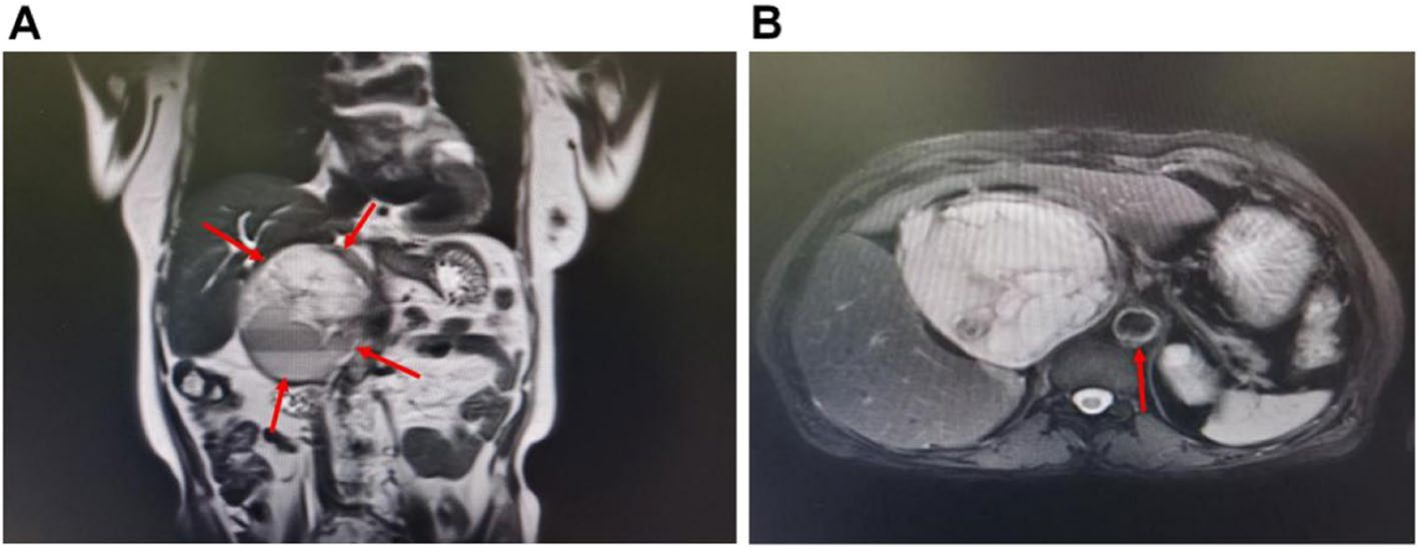
**A**, contrast-enhanced MRI scan showing a heterogenous mass of 11 cm in diameter in the right retroperitoneal region (arrows). **B**, contrast-enhanced MRI scan showing the giant mass pressing against the surrounding blood vessels; only the abdominal aorta is clearly visible (arrows); an axial view

The echocardiographic scan indicated segmental ventricular wall movement abnormalities, moderate pulmonary hypertension, and only 38% ejection fraction (EF). Laboratory examination results showed that the levels of troponin I were 655.0 pg/mL (reference range 0–14), and of the N-terminal fragment of the prohormone brain natriuretic peptide (NT-proBNP) 3930.0 pg/mL (reference range 0–300). The patient was preliminarily diagnosed with acute myocardial infarction and referred to the cardiac care unit (CCU). Emergency CAG examination was performed immediately and the result showed that there were no abnormalities in the coronary arteries and that myocardial ischemia was caused by a coronary spasm. Emergency computed tomography showed a uneven density mass with a dense nodular shadow (size, 106 × 106 × 128 mm) in the right retroperitoneal region, and magnetic resonance imaging(MRI)examination showed a giant cystic solid mass lesion in the right retroperitoneal with a size of 108 × 108 × 130 mm (Fig. 1). The patient’s diagnosis was confirmed using the free dopamine serum levels of 326.7 pmol/L (reference range ≤ 195.7), norepinephrine level of 5274.5 pmol/L (reference range 414–4435), and epinephrine level of 5916.9 pmol/L (reference range ≤ 605.4). Based on the clinical symptoms, laboratory tests, and MRI examination, the patient was eventually diagnosed with pheochromocytoma and catecholamine cardiomyopathy. The multidisciplinary team examined the genes associated with PPGLs. Fortunately, no mutated genes were found, and the patient had no family history of PPGLs.

### Preparation before operation

The woman was orally treated with terazosin 2 mg, nifedipine 30 mg and metoprolol 47.5 mg each day. On day 6 of admission, the patient's cardiac function improved significantly, she was also given intravenous infusions of hydroxyethyl starch 1000 mL and crystal liquid 1500 mL until the day of the operation. To maintain a fasting blood glucose between 5.0 and 11.0 mmol/L, 5U insulin aspartate was injected subcutaneously in the morning, 3U at noon, and 3U at night.

After 18 days of careful management, the patient ceased to feel symptoms of palpitations and chest tightness, the levels of troponin I dropped to 45.0 pg/mL and NT-proBNP 269.1 pg/mL, her cardiac function significantiy improved, the echocardiographic scan indicated the wall thickness of each ventricle was normal, movement coordination and contraction amplitude were normal, EF improved to 68%, and the patient was referred to the department of general surgery for the pheochromocytoma resection.

**Fig. 2:**
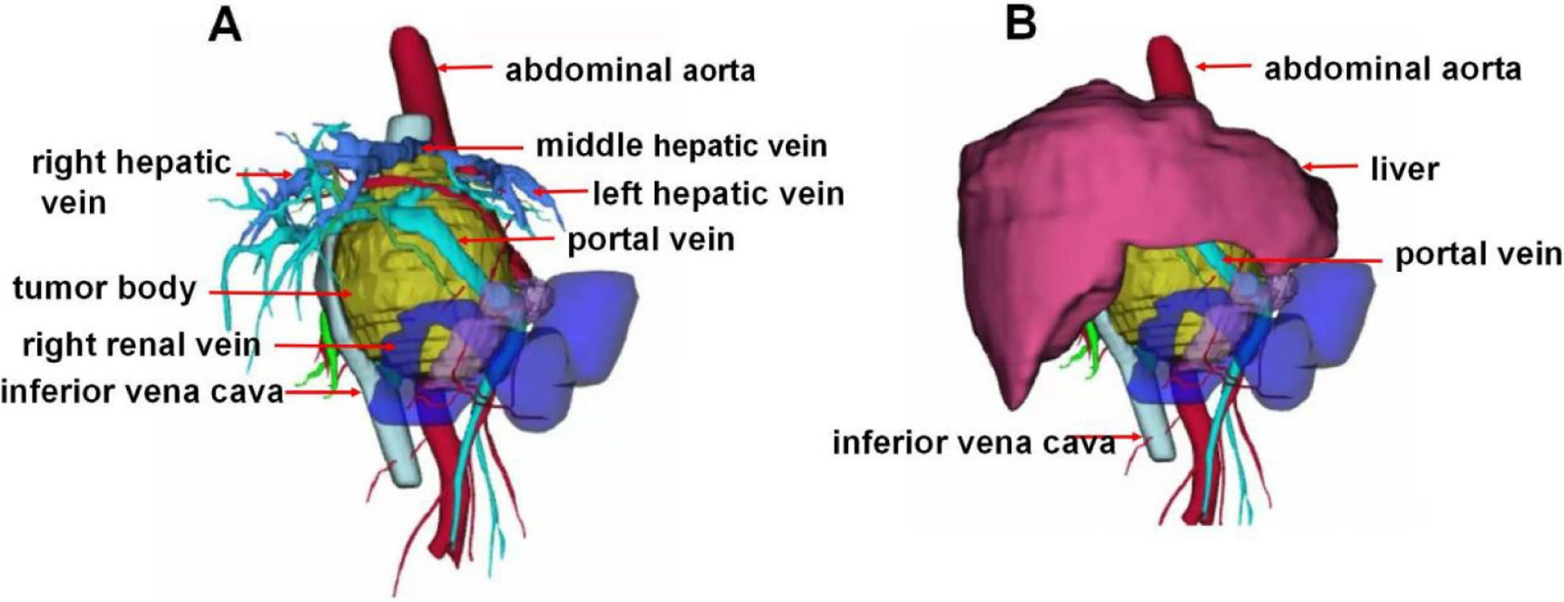
CT angiography and three-dimensional reconstruction shows the relationship between the tumor and abdominal vessels; the vessels surrounding the tumor are closely adhered to the tumor. **A**, liver removal. **B**, tumor body compressed portal vein and inferior vena cava

Computed tomography ( CT) angiography and a three-dimensional reconstruction of the abdominal vessels showed that the celiac trunk, common hepatic artery, gastroduodenal artery, right renal artery, portal vein, splenic vein, inferior vena cava, superior mesenteric vein, hepatic vein and right renal vein were compressed by the mass (Fig. 2).

### Preparation before anesthesia

Nearly 2 weeks later, a multidisciplinary team assessed the patient's whole condition, and especially an anesthesiologist team assessed the cardiac function and endocrine status, and the team considered that it was the appropriate time for the patient to undergo an open laparotomy and pheochromocytoma resection.

### Anesthesia method and medication

After the patient’s arrival at the operating room, ECG, oxygen saturation were monitored, invasive arterial blood pressure (IBP) was monitored after radial artery cannulation, and central venous pressure (CVP) was monitored via a central venous catheter after an internal jugular vein puncture with 1% lidocaine local anesthesia. The initial IBP was 136/82 mmHg, CVP was 6.0 cmH_2_O, HR was 92 beats/min and SpO_2_ was 96% under room air. The following anesthetics for induction were injected via the central venous catheter, midazolam 4.0 mg, sufentainl 30 μg, cisatracurium 10 mg, etomidate 16 mg, and dexmedetomidine 12 μg. Tracheal intubation was performed with a reinforced tracheal tube after two minutes of manual positive pressure ventilation. Anesthesia was maintained during the surgery via an intravenous infusion of propofol 4–6 mg/kg/hour, remifentanil 0.1–0.2 μg/kg/minute, and dexmedetomidine 0.5 μg/kg/hour. The initial arterial blood gas analysis showed that blood glucose was 12.6 mmol/L, PH:7.39, PaO_2_:368 mmHg, PaCO_2_:48 mmHg, SaO_2_:100%, HCT: 34, K ^+^: 4.6 mmol/L, Na^+^: 142 mmol/L, Ca^2+^:1.20 mmol/L, Lac: 3.0 mmol/L, Glu:12.6 mmol/L, and BE:-2.5 before the surgery.

### Circular management

**Fig. 3:**
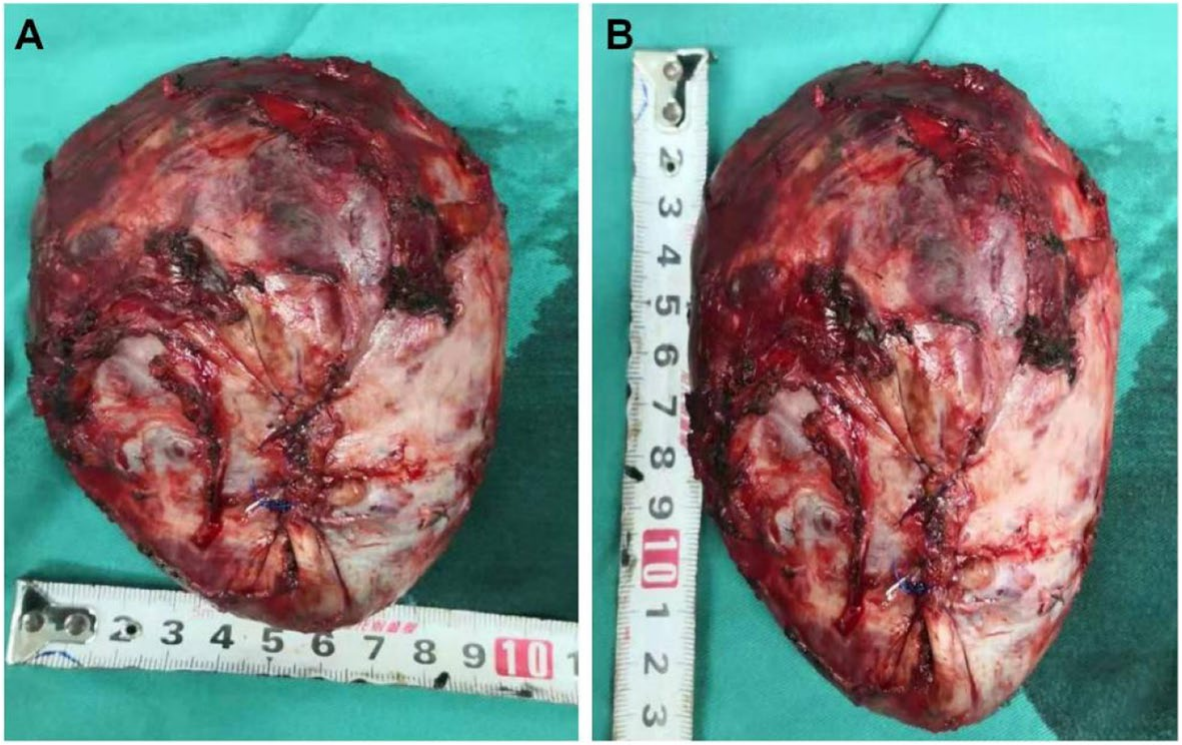
The image of resected giant tumor; 200 mL of cystic fluid was expelled during the operation, and immunohistochemical examination confirmed a completely excised paraganglioma. **A**: the width of 10.0 cm, **B**: the length of 13.0 cm

At the beginning of the surgery, the maintenance of haemodynamic stability using nitroglycerin 0.02 µg/kg/minute and esmolol 100 µg/kg/minute was successful. During the operative process, when the surgeons carefully probed and separated the tumor from the surrounding tissue, the IBP extremely fluctuated, with a peak of 205/112 mmHg and HR of 122 beats/minute. Thus, an intravenous infusion of sodium nitroprusside 0.2–3.0 μg/kg/minute, nitroglycerin 0.5–3.0 μg/kg/minute, and esmolol 50–500 μg/kg/minute was used to control the IBP between 160–120/60–100 mmHg. Phentolamine 2.0 mg was also intermittently intravenously injected to reverse the unstable IBP. At the same time, an arterial blood gas analysis showed that blood glucose was increased to 19.3 mmol/L, and so 10 U/hour insulin aspartate intravenous infusion was used to control blood glucose between 7.0–12.0 mmol/L. Blood gas analysis was then performed hourly (pH: 7.38–7.24, PaO_2_: 245–410 mmHg, PaCO_2_: 41–58 mmHg, SaO_2_: 99–100%, haematocrit (HCT): 29–35, K^+^: 3.2–5.6 mmol/L, Na^+^: 136–144 mmol/L, Ca^2+^: 1.13–1.26 mmol/L, Lac: 2.8–6.2 mmol/L, Glu: 19.3–11.2 mmol/L, and BE: -2.5– -13.0), and these related parameters were adjusted to the approximately normal range. The IBP dramatically dropped to 68/45 mmHg when the veins around the tumor were lapped and the tumor was resected (shown in Fig. 3). Intravenous infusions of norepinephrine 0.1–0.5 μg/kg/minute and dopamine 5.0–20.0 μg/kg/minute were used to control the IBP between 120–80/60–70 mmHg, fluid infusion was accelerated, and a total of approximately 7000 mL was administered with an equal ratio of crystal and colloid. Hydrocortisone 100 mg was injected to prevent an adrenergic-related cortisol crisis. After tumor resection, during hypotension, BE and lactic acid increased gradually, reaching a peak of -12.4 and 8.3 mmol/L, respectively. Continuous intravenous infusion of 250 mL 5% sodium bicarbonate was performed. According to blood gas analysis results, the amount of sodium bicarbonate was increased, and finally BE and lactic acid decreased to -4.2 and 3.2 mmol/L, respectively. In summation, the duration of the surgery was totally 8.5 h with a total blood loss of 2500 mL, urine production of 1200 mL, and transfusion of 1050 mL of red blood cells, 900 mL of fresh frozen plasma, 3500 mL of hydroxyethyl starch, and 3500 mL of lactated Ringer's solution. The patient was transferred to the intensive care unit (ICU) after surgery.

### Patient outcome

**Fig. 4:**
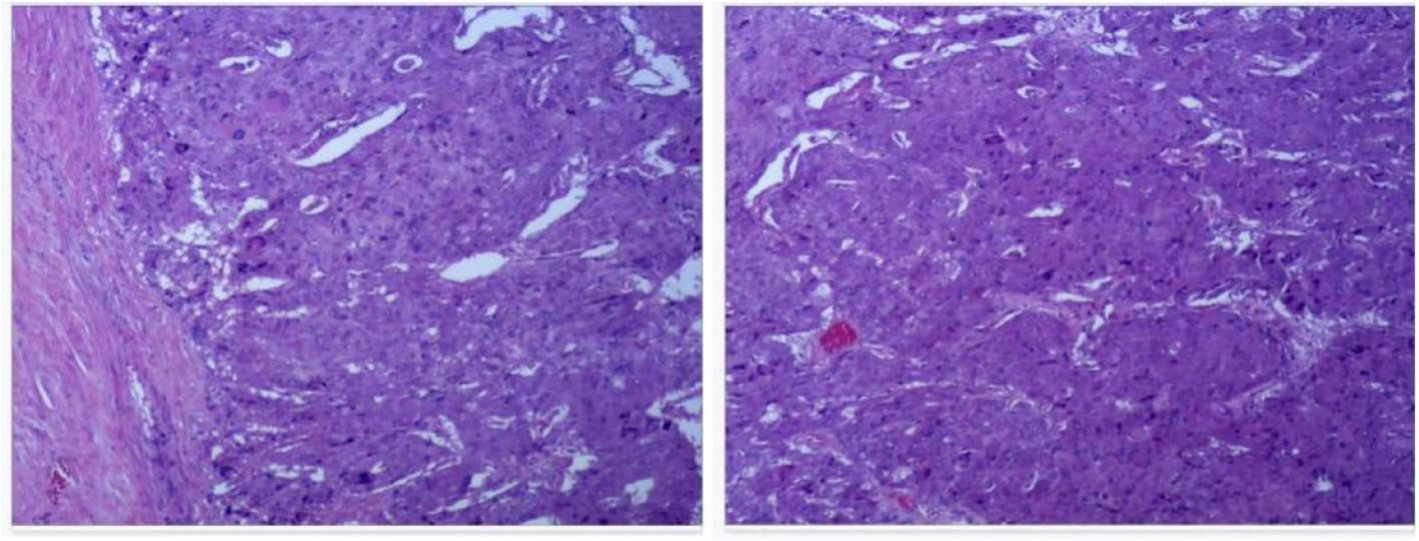
Pathological diagnosis of retroperitoneal paraganglioma. Under an optical microscope, the tumor cells showed obvious nests and organoid structures (Zellballen). The tumor cells are polygonal, granular and dichroic. Flattened S-100 positive sertoli cells are seen around nests of tumor cells. Immunohistochemistry shows CgA ( +), S-100 ( +), and Syn ( +)

The endotracheal intubation was removed 18 h after surgery, and oxygen was supplied via mask inhalation of 2 L/minute with SpO_2_ > 98%. The ICU physicians complained that the patient had obvious systemic edema, which was significantly improved 24 later at 2000 mL dehydration and diuresis treatment, and after diuresis 3000 ml on the second and third day after surgery, the symptom of systemic edema disappeared completely. The vasoactive drugs were gradually reduced while the patient was in the ICU, and no vasoactive drugs were required 48 h after the surgery as the patient’s hemodynamics remained stable. A laboratory biochemical test showed that the levels of troponin I and NT-proBNP dropped to 66 pg/mL and 348 pg/mL, respectively. Postoperative pathology and immunohistochemical examination confirmed the diagnosis of paraganglioma with a size of 108 × 108 × 130 mm and weight of 1100 g (Fig. 4). The patient was discharged after 45 days of hospitalization, with normalized serum levels of free dopamine, norepinephrine, and epinephrine.

## Discussion

Common clinical symptoms of PPGLs include hypertension, typical headache, hypersweating, palpitations, anxietys and tremors, which, if left untreated, can increase a patient's risk of cardiovascular disease and even lead to death. The patient presented with palpitations, nausea, and vomiting as the initial symptoms, and the patient's family reported that her mother often presented with palpitations and dyspnea for more than 10 years. Combined with the ST segment elevation of 0.8 mV and myocardial enzyme spectrum results showed that troponin I increased to 655 pg/mL and NT-proBNP, 3930.0 pg/mL, the patient was initially diagnosed with acute myocardial infarction. However, emergency coronary angiography showed no abnormalities in the coronary arteries. Subsequent CT and MRI revealed a giant retroperitoneal mass, and biochemical results showed abnormally elevated catecholamine levels. The patient was eventually diagnosed with ectopic pheochromocytoma and catecholamine cardiomyopathy. Postoperative pathology and immunohistochemistry confirmed paraganglioma.

Considering that 70–80% of patients with PPGLs have genetic factors (including 20 gene mutations), the main mutated genes are related to hypoxia stress, kinase signal transduction, and protein translation [6]. If the mutated gene in a patient with PPGL can be identified, the nuclear physicians can develop a precise radiotherapy regimen, which is of great benefit to the patient [7]. The multidisciplinary team examined 10 of the patient’s genes (MAX、NF1、RET、SDHAF2、SDHA、SDHB、SDHC、SDHD、TMEM127、and VHL) that were associated with PPGLs. Fortunately, no mutated genes were found, and the patient had no family history of PPGLs. In addition, therapeutic strategies for specific genetic mutations have been proposed, but these strategies are still too underdeveloped to be applied clinically [8].

There are many surgical methods for PPGLs. Due to the need to establish a pneumoperitoneum, the laparoscopic-assisted resection of PPGLs was previously considered to be dangerous but is now the preferred technique, especially for PPGLs with a diameter < 6 cm. Laparoscopic-assisted tumor resection has the advantages of more stable perioperative haemodynamics, faster postoperative recovery, and fewer complications; however, open surgery is recommended for large PPGLs (> 6 cm) [2, 9–11]. The patient underwent cholecystectomy in another hospital 10 years prior, and abnormally increased blood pressure was found during the operation, but the cause was not identified. At the beginning of this operation, laparoscopic exploration yielded a serious adhesion between the tumour and the surrounding organs and vessels; thus, laparoscopic surgery was changed to open surgery.

Adequate preoperative preparation and accurate perioperative management are the keys to reducing the mortality of PPGLs. Previous data indicated that the mortality rate of those with PPGLs was approximately 40% [12, 13]. However, the mortality rate of those with PPGLs is now reduced to 0–3% in a multidisciplinary team in a specialized medical center [14, 15]. The improved prognosis of those with PPGLs can be attributed to the optimization of preoperative antihypertensive drugs and advances in modern imaging technology, including angiography and three-dimensional vascular reconstruction, which can accurately locate the tumor location and help determine the relationship between the tumor and surrounding blood vessels. In order to prevent hypotension after PPGL resection, previous studies have recommended a high-salt diet and 2000 mL of saline supplementation 24 h before surgery [16, 17], but the evidence now seems limited. A perioperative transesophageal ultrasound can be used to observe the degree of ventricular filling to determine volume deficiency and used an echocardiogram to determine cardiac function [18], which has also been challenged [19].

The regulation of hyperglycemia is very important for the perioperative management of PPGLs patients. PPGLs promotes the secretion of catecholamines, and high levels of catecholamines can lead to insulin resistance and decreased insulin secretion, resulting in hyperglycemia [20]. Approximately 15–35% of patients with PPGLs have impaired glucose tolerance and diabetes mellitus [21, 22]. Approximately 60.86% of patients had PPGLs combined with glucose tolerance impairment and diabetes mellitus before surgery, and tumor size was positively correlated with glucose tolerance impairment. The average size of patients with PPGLs combined with glucose tolerance impairment was 74.66 ± 11.98 mm, and 71.42% of these patients had improved glucose tolerance impairment after PPGL resection [23].

The patient’s fasting blood glucose was 10.7 mmol/L on the second day of admission and 21.0 mmol/L after breakfast; the patient denied a family history of diabetes. After careful management by the endocrinologist, the patient's fasting blood glucose was 7.5 mmol/L on the day before the surgery and 9.0 mmol/L after breakfast. During the operation, the highest blood glucose reached 19.2 mmol/L and dropped to 10.2 mmol/L after continuous intravenous infusion of insulin at 10 U/hour. Blood glucose returned to normal on the third day after surgery, and there was no abnormality in the blood glucose during the half-year follow-up.

The prevention of hypotension after tumor resection in patients with PPGLs is important for the improvement of patient outcomes, and adequate preoperative preparation is critical. Blood pressure, orthostatic hypotension, and electrocardiograms are recommended by the Endocrine Society Practice Guidelines as important parameters for preoperative preparation [24]. Patients with pheochromocytoma and diabetes, cardiovascular and cerebrovascular diseases, preoperative elevated blood glucose levels, increased catecholamine levels, and pheochromocytoma > 5 cm are all risk factors for hypotension after tumor resection, as well as high risk factors for postoperative complications [25]. The patient’s IBP dramatically dropped to 68/45 mmHg when the tumorous was resected. We also observed that the patient required larger doses of vasoconstrictors and that they needed to be administered more frequently. Using an intravenous infusion of norepinephrine 0.1–0.5 μg/kg/minute and dopamine 5.0–20.0 μg/kg/minute and an intermittent intravenous infusion of norepinephrine 40 μg the IBP was maintained between 120–80/60–70 mmHg. When the surgeon closed the abdominal cavity, the patient was found to have obvious systemic edema. Our analysis suggested that norepinephrine was diluted to 2ug/mL by normal saline, and a large amount of normal saline was brought into the body with a large dose of norepinephrine, resulting in edema. Under the condition of ensuring sufficient fluid of the patient, after diuresis of 3000 mL, the patient's edema was significantly improved on the second day, and after 3000 ml diuretic on the third day, the edema symptom disappeared completely. Thus, we concluded that high concentrations of norepinephrine diluted with saline may prevent systemic edema.

The multidisciplinary team developed a detailed preoperative preparation strategy for the patient, including preoperative changes in body weight, HCT, and HR as important indicators of adequate preoperative preparation [26, 27]. Preoperative systolic blood pressure < 130 mmHg and/or diastolic pressure of 85 mmHg, weight gain of > 2%, HR of 60 to 100 beats/minute, and HCT of 25% to 45% may reduce the incidence of perioperative hypotension [28]. After our adequate preoperative preparation, the patient’s body weight increased by 4% (from 48 to 50 kg), her HR decreased from 142 beats/minute on admission to 82 beats/minute on the day before the surgery, and her HCT was 35.1%. All of these strategies provided a good guarantee for the patient to safely survive the perioperative period.

## Conclusions

Patients with PPGLs usually initially present with palpitations, chest tightness, and headaches, and are thus easily diagnosed with cardiovascular disease. Subsequent CT or MRI examinations and biochemical tests can be used to easily confirm the presence of PPGLs. Most PPGLs can be cured via surgical excision. Many factors, such as large PPGLs, long history of PPGLs, and preoperative glucose tolerance abnormalities, have been closely associated with postoperative survival. Adequate preoperative preparation, accurate perioperative management and careful postoperative monitoring are important strategies to improve the prognosis of patients with PPGLs. In recent years, the discovery of PPGL-related genes has contributed to great progress in the diagnosis and treatment of malignant PPGLs. The goal of future research is to clarify the tumor-inducing mechanism of PPGL gene mutations and provide effective targeted therapies for the early diagnosis of PPGLs and the development of targeted drugs.

## Data Availability

All data generated or analysed during this study are included in this published article.

## Abbreviations

AMI: Acute myocardial infarction
MAP: Mean arterial blood pressure
HR: Heart rate
PPGLs: Pheochromocytomas and paragangliomas
NIBP: Noninvasive blood pressure
ECG: Electrocardiography
SpO_2_: Peripheral capillary oxygen saturation
MRI: Magnetic resonance imaging
EF: Ejection fraction
CT: Computed tomography
CAG: Percutaneous coronary angiography
CCU: Cardiac care unit
IBP: Invasive arterial blood pressure
CVP: Central venous pressure
BNP: Brain natriuretic peptide
